# Investigating the efficacy and importance of mobile-based assessments for Parkinson's disease: uncovering the potential of novel digital tests

**DOI:** 10.1038/s41598-024-55077-7

**Published:** 2024-03-04

**Authors:** Yanci Zhang, Zhiwei Zeng, Maryam S. Mirian, Kevin Yen, Kye Won Park, Michelle Doo, Jun Ji, Zhiqi Shen, Martin J. McKeown

**Affiliations:** 1 School of Computer Science and Engineering, Nanyang Technological University, Singapore, Singapore; 2 Joint NTU-UBC Research Centre of Excellence in Active Living for the Elderly, Nanyang Technological University, Singapore, Singapore; 3 Pacific Parkinson’s Research Centre, University of British Columbia, Vancouver, Canada

**Keywords:** Parkinson's disease, Health services, Diagnostic markers

## Abstract

This study introduces PDMotion, a mobile application comprising 11 digital tests, including those adapted from the MDS-Unified Parkinson's Disease Rating Scale (MDS-UPDRS) Part III and novel assessments, for remote Parkinson's Disease (PD) motor symptoms evaluation. Employing machine learning techniques on data from 50 PD patients and 29 healthy controls, PDMotion achieves accuracies of 0.878 for PD status prediction and 0.715 for severity assessment. A post-hoc explanation model is employed to assess the importance of features and tasks in diagnosis and severity evaluation. Notably, novel tasks that are not adapted from MDS-UPDRS Part III like the circle drawing, coordination test, and alternative tapping test are found to be highly important, suggesting digital assessments for PD can go beyond digitizing existing tests. The alternative tapping test emerges as the most significant task. Using its features alone achieves prediction accuracies comparable to the full task set, underscoring its potential as an independent screening tool. This study addresses a notable research gap by digitalizing a wide array of tests, including novel ones, and conducting a comparative analysis of their feature and task importance. These insights provide guidance for task selection and future development in PD mobile assessments, a field previously lacking such comparative studies.

## Introduction

Parkinson’s disease (PD) is one of the most common neurodegenerative diseases. In 2016, 6.1 million people were living with PD worldwide, and the disease caused more than 0.2 million deaths^1^. In 2020, the number of people living with PD had grown to 9.4 million^2^. This long-term neurological disorder leads to a progressive deterioration of symptoms, especially in motor functions. Commonly occurring motor symptoms of PD include tremors, bradykinesia, stiffness, rigidity and difficulty in walking, balance and coordination. Frequent monitoring can help with screening the disease in its early stages in those who have prodromal symptoms of the disease or risk factors (e.g., rapid eye movement sleep behavior disorders, family history of PD, etc.). In addition, systematic evaluation of motor symptoms can help guide clinicians in medical management and medication adjustment.

With the growing popularity of mobile phones, remote disease assessments via mobile phones offer a convenient and non-intrusive way of detecting incipient signs and monitoring the progression of PD. As an interactive data collection tool, mobile phones can collect a rich variety of data that may help to characterize symptoms of PD. For example, motion data can be collected from embedded accelerometers and spatiotemporal data of finger movement can be collected from screen interactions. Benefiting from the increasing popularity of mobile phones, researchers have designed digital assessment tasks on mobile phones to capture characteristic features of PD motor symptoms. Some researchers focus on quick screening for PD by designing a single screening task. For example, in Ref.^3^, the authors designed a spiral tracing test and extracted geometric features to screen PD. Some other researchers focus on the assessment and severity evaluation of PD by combining multiple tasks to collaboratively detect different PD symptoms. Researchers in Ref.^4^ digitalized both a spiral drawing test and a tapping test to quantify the dexterity of PD. The authors in Ref.^5^ designed tests targeting the hand tremor, walking and turning motion on a mobile-based PD assessment and monitoring system. Similar platforms included mPDS^6,7^, which leveraged 5 tasks (voice, finger tapping, gait, balance, and reaction time) to assess PD motor symptom severity.

However, compared to the traditional PD assessment conducted physically in clinical settings, the understanding of different mobile motor assessments is still very limited. Although studies have been conducted to validate the effectiveness of individual tasks or a combination of tasks, there have been no comparative studies on the effectiveness of different tasks to guide the selection of tasks or the future design of mobile assessments of PD.

To fill in this gap, we built PDMotion, a mobile platform for automatic PD motor examination, and studied its effectiveness in accurately distinguishing PD patients and differentiating various severity levels of PD patients from healthy controls (HCs). From a machine learning perspective, we also seek to study and gain a deeper understanding of different mobile motor assessments by analyzing their importance in diagnosis and severity evaluation. Our research uniquely contributes to the field of PD assessments by designing and evaluating a comprehensive range of digital tests, including novel digital assessments that are not adapted from traditional PD motor evaluations. We also present an extensive comparison between the novel digital assessments and those adapted from traditional paper-and-pencil tests. A key aspect of our work is the analytical focus on identifying the most important tests for predicting PD status and severity within mobile health applications. We discovered that tasks not derived from MDS-UPDRS Part III play a significant role, indicating that digital assessments for PD need not be limited to the mere digitalization of existing tests. Notably, our findings also suggest the alternative tapping test's potential as an effective standalone screening tool, providing valuable insights for future task selection and development in PD mobile assessments.

**Figure 1:**
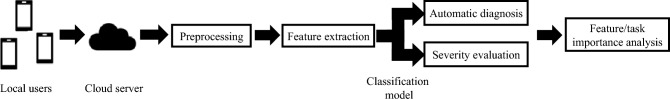
PDMotion pipeline.

As illustrated in Fig. 1, the pipeline of PDMotion is divided into four steps: (1) application data collection, (2) feature extraction, (3) automatic diagnosis and severity evaluation, and (4) feature/task importance analysis. In the application data collection step, PDMotion collects motion and spatiotemporal data from built-in accelerometers and on-screen interactions while the participant is engaging with it. Feature extraction involves extracting representative features from collected data, guided by medical criteria. Automatic diagnosis and severity evaluation are achieved by applying machine learning-based classification models to the extracted features. Lastly, feature/task importance analysis is performed using a model-agnostic, post-hoc explanation model on the classifier.

## Results

### Test subject description

As shown in Table 1, 79 subjects were recruited at the Pacific Parkinson’s Disease Research Centre to test PDMotion, including 50 patients with PD and 29 healthy controls.

**Table 1:** Dataset characteristics.

	PD group (n = 50)	HC group (n = 29)
Demographic		
Age (years)		
Mean (SD)	67.58 (9.23)	69.00 (7.25)
Min/max	43/82	55/88
Gender		
Female	18	15
Male	32	14
Clinical		
MDS-UPDRS Part III score		
Mean (SD)	32.00 (12.51)	9.10 (6.91)
Min/max	8/68	0/31
Mild/moderate/severe	25/24/1	N/A
Hoehn &Yahr stage		
Mean (SD)	1.94 (0.71)	N/A
Min/max	1/5	N/A
1/2/3/4/5	10/36/2/1/1	N/A
MoCA score		
Mean (SD)	25.86 (3.10)	25.90 (3.18)
Min/max	18/30	16/30

Among the 50 PD patients (the PD group), 18 were female and 32 were male. The ages of the patients ranged from 43 to 82, with an average age of 67.58 years old (standard deviation (SD) 9.23). Their MDS-UPDRS Part III scores ranged from 8 to 68, with an average score of 32.00 (SD 12.51). Based on the MDS-UPDRS Part III scores, 25 patients were at a mild level (score ≤ 32), 24 were at a moderate level (33 ≤ score ≤ 58), and 1 patient was at a severe level (score ≥ 59)^8^. The Hoehn and Yahr (H&Y) stages of the patients varied from 1 to 5, and the average H&Y stage was 1.94 (SD 0.71). Specifically, there were 10 patients at stage 1, 36 patients at stage 2, 2 patients at stage 3, 1 patient at stage 4 and 1 patient at stage 5. The Montreal Cognitive Assessment (MoCA) scores of the patients ranged from 18 to 30, with an average of 25.86 (SD 3.13). In our study, participants with a MoCA score below 16 were excluded due to the high probability of cognitive impairment in these individuals, which could adversely impact their performance on tasks. Diminished performance resulting from cognitive impairments could be erroneously attributed to motor symptoms of PD and compromise the accuracy of our predictive model.

For the healthy control group (the HC group), 15 were female and 14 were male. The ages of the healthy participants ranged from 55 to 88, with an average age of 69.00 years old (SD 7.25). For better comparison with the PD group, MoCA and MDS-UPDRS Part III were also administered for the HC group before conducting tests on the PDMotion. The MDS-UPDRS Part III scores of the HC group ranged from 0 to 31, with an average score of 9.10 (SD 6.91). It is noteworthy that elevated MDS-UPDRS scores are frequently observed in the general population without parkinsonism due to the effect of age, gender, and/or comorbidity conditions^9^. The MoCA scores of HCs ranged from 16 to 30, with an average score of 25.90 (SD 3.18). The same exclusion criterion based on the MoCA score was applied to the recruitment of HCs. There was no statistically significant difference between the cognitive functionalities of the PD and HC groups (*p*-value: 0.958). Since the H&Y examination was designed for evaluating the severity stages of PD, it was not administered for the HC group. We regarded their H&Y stages as 0 during experiments.

**Figure 2:**
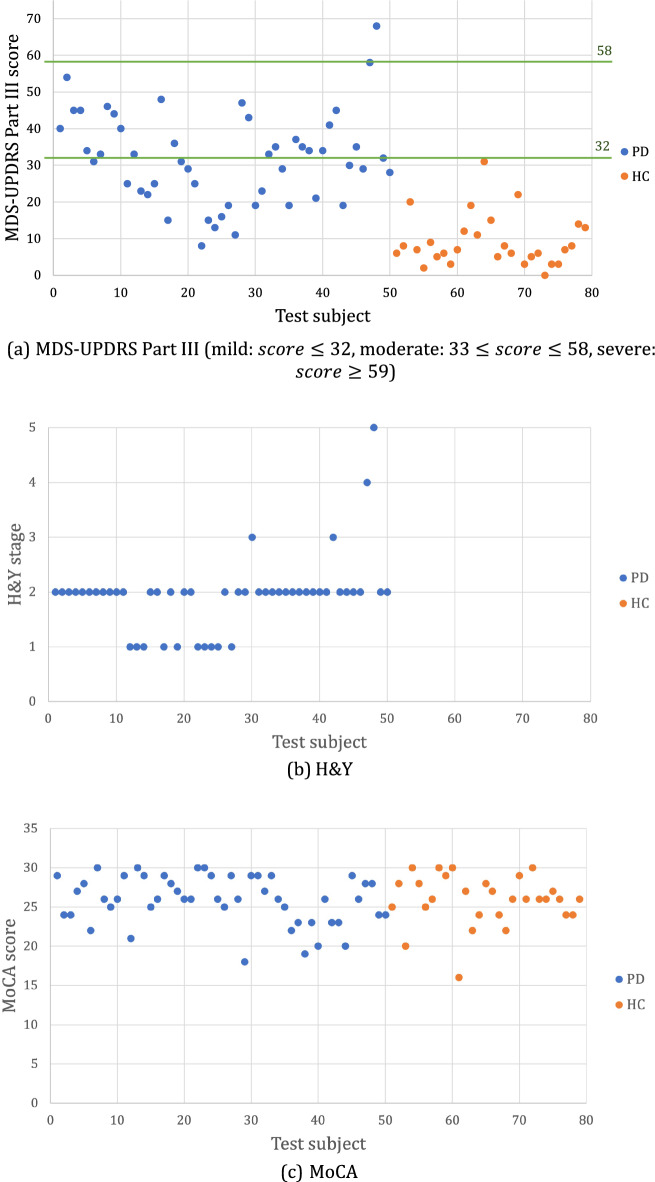
Distribution of test subjects under the three medical rating scales.

Figure 2 shows the distribution of all test subjects under the three medical rating criteria, MDS-UPDRS Part III, H&Y and MoCA.

### Diagnosis and severity evaluation results

The train-test split used for the classification task was 9:1, 90% for training and 10% for testing. For each model, we conducted the split-train-test process 100 times, performed a grid search to identify the optimal parameter settings, and then calculated the average evaluation metrics on the test sets.

The classification models predict class labels based on the extracted features. We used the accuracy, as well as the weighted average precision, recall, and F1 score of all classes to evaluate the performance of the classification models. The accuracy is calculated by:

$$Accuracy=\frac{{N}_{c}}{{N}_{t}}$$

where ${N}_{c}$ refers to the number of correct predictions regardless of predicted classes or ground truth classes, and ${N}_{t}$ refers to the number of total predictions. The weighted average precision, recall, and F1 score are calculated following:

$$W= \frac{\sum_{i=1}^{n}{w}_{i}{X}_{i}}{\sum_{i =1}^{n}{w}_{i}}$$

where ${w}_{i}$ is the number of test subjects per class, and ${X}_{i}$ is the test performance (precision, recall, and F1 score) for each class. Weighted averages were used because the classes are highly imbalanced. The results are shown in Table 2.

**Table 2:** Classification results.

Classification criteria	Linear neural network	SVM	Random forest	XGBoost	Logistic regression
PD/HC 2 classes: HC, PD					
Accuracy	0.809 (0.138)	0.842 (0.126)	0.817 (0.099)	**0.878 (0.116)**	0.840 (0.131)
Precision	0.826 (0.136)	0.862 (0.111)	0.818 (0.107)	**0.853 (0.152)**	0.856 (0.126)
Recall	0.802 (0.142)	0.839 (0.143)	0.816 (0.104)	**0.860 (0.142)**	0.824 (0.158)
F1 score	0.792 (0.141)	0.829 (0.136)	0.801 (0.106)	**0.841 (0.150)**	0.817 (0.151)
H&Y 6 classes: HC, stage 1–5					
Accuracy	0.575 (0.177)	0.689 (0.168)	0.682 (0.151)	**0.715 (0.140)**	0.658 (0.162)
Precision	**0.487 (0.241)**	0.371 (0.217)	0.423 (0.214)	0.467 (0.233)	0.477 (0.237)
Recall	0.471 (0.206)	0.446 (0.199)	0.464 (0.237)	**0.520 (0.236)**	**0.520 (0.209)**
F1 score	0.454 (0.208)	0.388 (0.203)	0.427 (0.218)	**0.473 (0.224)**	0.472 (0.215)
MDS-UPDRS Part III 4 classes: HC, mild, moderate, severe					
Accuracy	0.560 (0.168)	**0.636 (0.170)**	0.476 (0.144)	0.570 (0.165)	0.628 (0.171)
Precision	0.521 (0.194)	**0.579 (0.217)**	0.460 (0.177)	0.528 (0.191)	0.557 (0.218)
Recall	0.506 (0.188)	**0.613 (0.212)**	0.494 (0.149)	0.539 (0.183)	0.600 (0.191)
F1 score	0.477 (0.176)	**0.555 (0.206)**	0.438 (0.143)	0.501 (0.174)	0.544 (0.198)

^a^Bold font suggests the best results under a certain classification and evaluation criterion.

In general, the model performances under three classification criteria can be ranked in descending order as PD/HC, H&Y, and MDS-UPDRS based on the classification results, regardless of the deployed machine learning models.

Firstly, it is observed that models performed better for the PD/HC criterion than H&Y and MDS-UPDRS Part III criteria. One possible reason may be that 2-class classification is less complex than multi-class classification by instinct.

Secondly, comparing the results of two multi-class classification tasks, the models performed better in the H&Y stage classification than in the MDS-UPDRS Part III severity classification task. This finding was beyond our expectations in two aspects. A large portion of tests in PDMotion was digitalized from MDS-UPDRS Part III questions. Therefore, we hypothesized that the prediction based on the collected PDMotion data could better capture the test subject’s performance related to MDS-UPDRS Part III. On the other hand, H&Y divided the participants into 6 classes, making the prediction intuitively more challenging than the 4-class classification of MDS-UPDRS Part III. The model achieved lower accuracy under MDS-UPDRS Part III criteria than H&Y criteria. The difference in performance may be attributed to the difference in the data labelling process. As described in the Test Subject Description, both HC and PD groups went through the MDS-UPDRS Part III and had valid MDS-UPDRS Part III scores. Many HCs had similar MDS-UPDRS Part III scores as patients with mild PD symptoms, making it harder to distinguish them. On the other hand, all HC patients do not have H&Y evaluations and were treated as H&Y stage 0.

Among all deployed machine learning models, XGBoost achieved the best overall performance. It achieved the highest performance in all metrics under the PD/HC classification criterion and the highest accuracy, recall, and F1-score under the H&Y classification criterion. XGBoost is a tree-based ensemble machine learning model that sequentially and additively trains a set of decision trees to make decisions. Tree-based models are genuinely immune to multi-collinearity. Since XGBoost is composed of boosted individual decision trees, it is also unaffected by multi-collinearity, making it perfectly suitable for our extracted features. As displayed in Table 2, XGBoost achieved an accuracy of 0.878 for binary classification based on HC/PD; 0.715 for 6-class classification based on H&Y stages; and 0.570 for 4-class classification based on MDS-UPDRS Part III scores.

### Feature & task importance analysis

**Figure 3:**
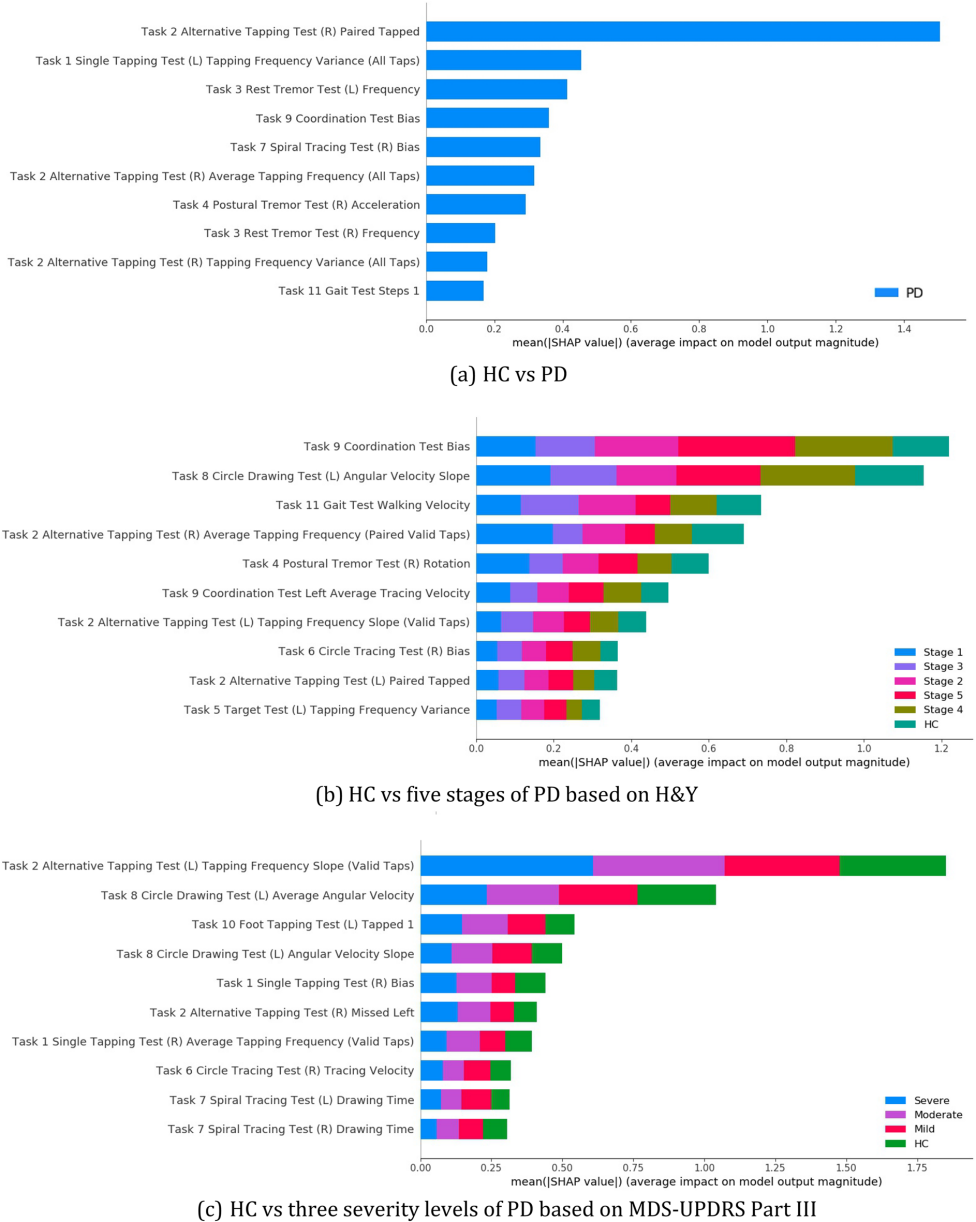
The top 10 features explained by SHAP for the three classification scales.

We generated the feature importance ranking by applying SHAP to the most accurate classification model, XGBoost. We calculated the Shapley value for each feature to evaluate its contribution to the decision-making process. The global importance of a feature is aggregated from every data point, by calculating the mean absolute value of the Shapley values for the feature over the whole sample. Figure 3 shows the 10 most important features ranked by SHAP under the three classification criteria.

We utilized bar charts to show the global summary of feature importance. The length of the bars represents the aggregated mean absolute Shapley values, which illustrates the magnitude of the average impact that a feature has on model output. For binary classification (PD/HC), if a feature has a positive effect in pushing the model output towards one class, it is equivalent to having a negative effect, which has the same magnitude as the positive one, in pushing the model output away from the other class. Hence, for binary classification, only the mean absolute Shapley value for the PD class is plotted. In multi-class classification (H&Y and MDS-UPDRS Part III), the magnitude of the average impact that a feature has on each class is visualized separately, indicated by different colors.

As illustrated in Fig. 3a, the paired taps in task 2 (alternative tapping test (R)) is the most important feature in distinguishing the PD group from the HC group. It records the number of paired taps of the index finger and middle finger. Tapping outside the circle area or tapping not following the correct alternating left–right order is not counted as paired taps. The paired tapping number could reflect the exactness of finger control and the speed of finger movement. Other features also contribute to the classification process, but at a significantly smaller magnitude than the paired taps in task 2 (alternative tapping test (R)).

As shown in Fig. 3b, for differentiating H&Y stages, the three most important features are extracted from three tasks from different task categories, the coordination test, the circle drawing test, and the gait test. Bias in task 9 (coordination test) is the most important feature as shown in Fig. 3b. It measures the difference between the user-drawn trail and the displayed trail. We hypothesized that the bias might be caused by both impaired motor ability to control finger movement and the coordination requirement of moving the index fingers of both hands simultaneously. It is easier to obtain this feature in digital tests than in traditional paper-and-pencil tests. The angular velocity slope in task 8 (circle drawing test (L)) is the second influential feature as shown in Fig. 3b, which is only slightly less important than the top feature. It measures the reduction of angular velocity when the test subject draws a circle as large as possible on the screen with his/her index finger. The slope is calculated by fitting a linear regression model on the angular velocity at every time stamp with an interval of 0.3 s. The slowing tendency of angular velocity could be triggered by the easy fatigue due to PD. It is worth noting that the coordination test and the circle drawing test were not a part of MDS-UPDRS Part III. However, research in Ref.^10^ provided evidence for impaired finger coordination in fine motor control of PD patients and research in Ref.^11^ included the circle drawing test to investigate micrographia.

For separating different levels of MDS-UPDRS motor abilities, the alternative tapping test and circle drawing test also have a significant influence on the classification results. The tapping frequency slope of valid taps in task 2 (alternative tapping test (L)) is shown as the top important feature as displayed in Fig. 3c. The valid taps refer to any tap within the restricted circle area, regardless of whether the taps are paired. The slope is calculated by fitting a linear regression model on the tapping frequency. It may reflect the early fatigue tendency and the impaired finger control ability of the test subjects. The average angular velocity in task 8 (circle drawing test (L)) also has a considerable effect on determining MDS-UPDRS Part III severity levels, but with less importance. It may indicate bradykinesia.

**Figure 4:**
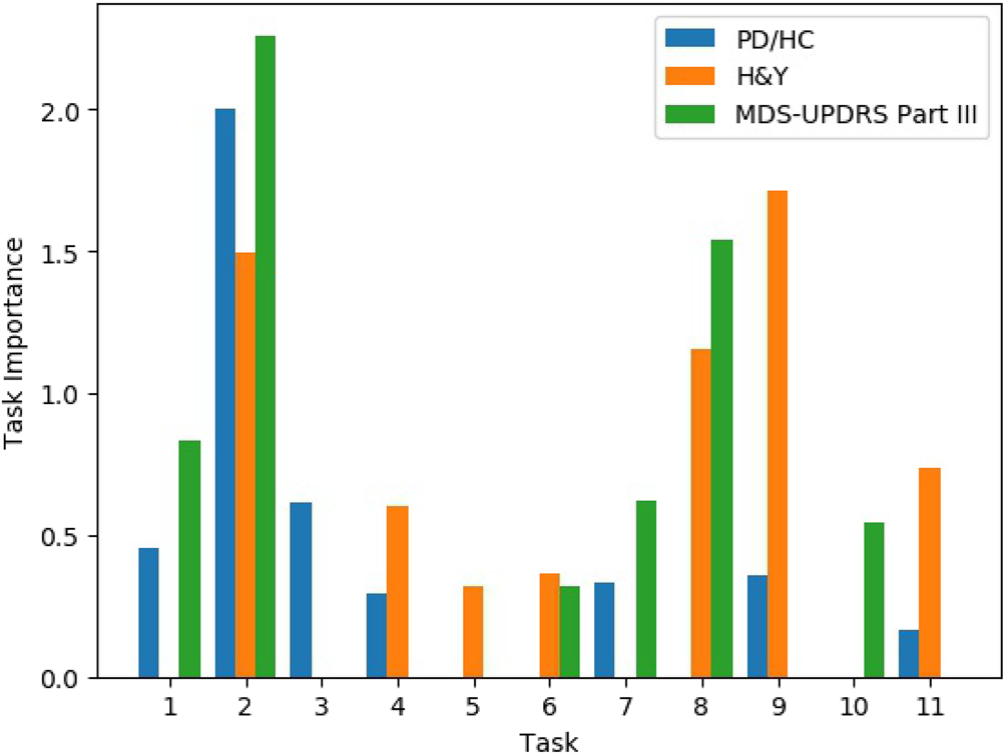
Task importance (Sum of top 10 feature mean absolute Shapley values).

We further analyze the SHAP results at the task level. Using the feature importance ranking list, the task importance is calculated as the sum of the absolute Shapley values of its features. As observed from Fig. 3, the effect size of the top 10 most important features decreases very fast. Hence, we only include the top 10 features in the task importance calculation. This is to avoid the case that a task with many unimportant features is ranked before a task with a few very important features. Figure 4 displayed the task importance for 11 tasks.

In general, it can be observed that task 2 alternative tapping, task 8 circle drawing, and task 9 coordination are more important for the diagnosis and severity evaluation tasks. Task 2 alternative tapping test has a significant influence on the results of all three classification tasks. It is the most important task for PD/HC classification and MDS-UPDRS Part III severity evaluation. For H&Y severity evaluation, it also has a high importance which is just smaller than task 9 coordination. Task 9 is the most important task for H&Y severity evaluation, while it does not have much influence on the results of the other two classification tasks.

Task 2 alternative tapping is digitalized from MDS-UPDRS Part III 3.4 tapping test. The high task importance suggested a successful digitalization of the tapping test on the mobile phone. Task 8 circle drawing and task 9 coordination are novel tests that do not have equivalents in the MDS-UPDRS Part III. Task 8 circle drawing test is designed to screen for micrographia. Task 9 coordination test is designed based on the research in Ref.^12^, which provided evidence for impaired finger coordination in fine motor control of PD patients.

As task 2 alternative tapping demonstrated very high importance in all classification tasks, we further studied its influence on the classification results with the experiment using only features from task 2 as inputs to classification models and comparing the results with that of using all features.

**Figure 5:**
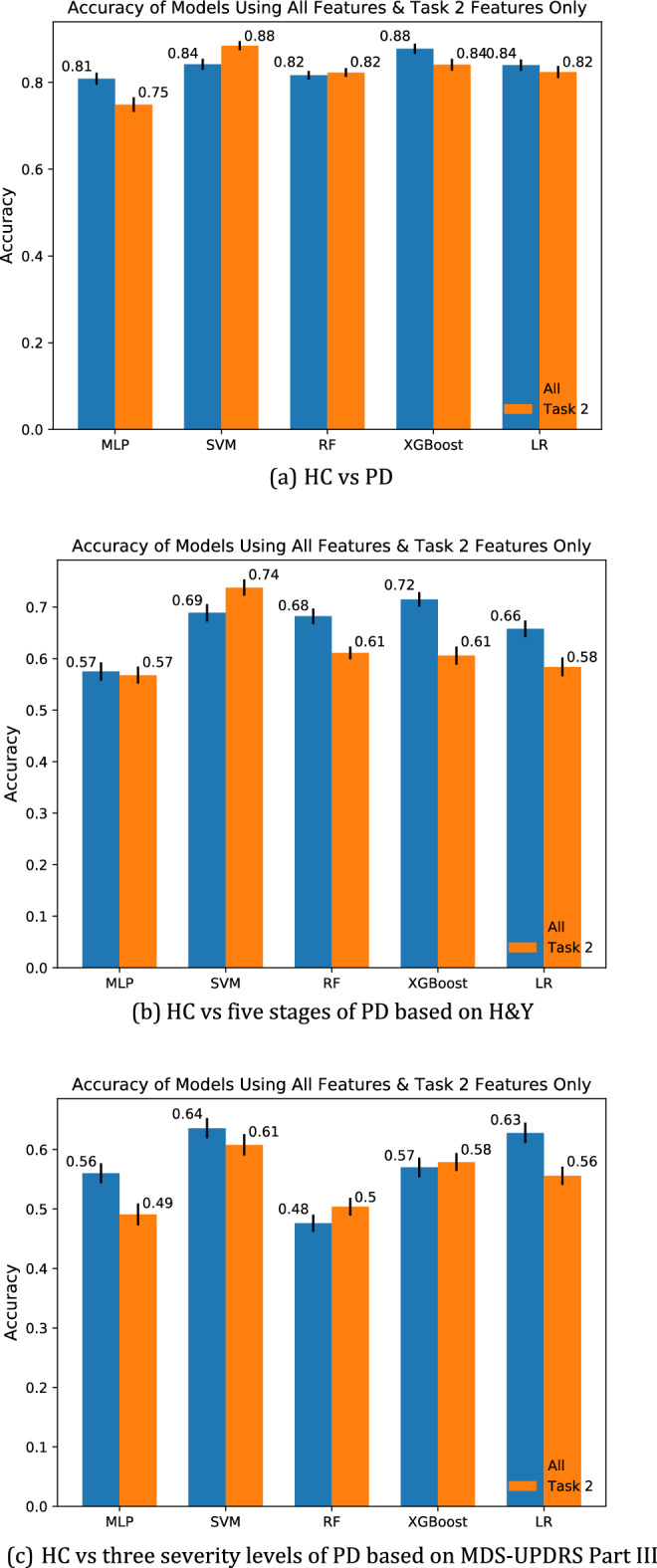
Comparison of model accuracy between using all features and using only task 2 features.

Figure 5 shows the results of the experiment. It illustrates the average accuracy with standard error (shown as the short black line at the top of the bar) for the results of all models in 100 runs on the test set. A few interesting observations can be drawn from the bar charts. Firstly, in general, the accuracies decrease by a small amount when only using features from task 2. However, there are also exceptions. For example, the SVM model yielded higher accuracies when only using task 2 features than using all features in HC vs PD (all: 0.84, task2_only: 0.88) and H&Y (all: 0.69, task2_only: 0.74) classification. Secondly, the magnitude of change (decrease/increase) in accuracies is larger in H&Y and MDS-UPDRS Part III than in HC vs PD classification. This is expected, as task 2 has a much larger influence on the results of HC vs PD classification as indicated by its much higher task importance under this classification criterion. Thirdly, instead of XGBoost, SVM is the best-performing model when only using task 2 features. This may be attributed to the fact that XGBoost is an ensemble model, hence its advantage is more prominent when there are more features and data.

## Discussion

Although many studies have investigated the possibility of applying digital tests to automatically diagnose PD through the detected motor symptoms, few have explored the importance of different digital tests in assessing PD motor ability from various perspectives. To the best of our knowledge, this is the first work investigating the importance of different motor examinations on a digital platform for detecting and monitoring the progression of PD. To evaluate the test importance, we designed and developed a comprehensive digital platform that involves 11 tests both focusing on PD symptoms covered in MDS-UPDRS Part III (e.g. tremors, finger and foot tapping, and gait) and other motor symptoms not directly covered in MDS-UPDRS Part III (e.g. micrographia and finger coordination). By utilizing machine learning techniques on the gathered digital data, the study showed that employing the 11 tests as a comprehensive kit can yield high prediction accuracy (accuracy of 0.878 (SD 0.116) for PD/HC; accuracy of 0.715 (SD 0.140) for H&Y; accuracy of 0.636 (SD 0.170) for MDS-UPDRS Part III scores). The accurate prediction serves as a robust basis for the reliability of analyzing the importance of tests. To further investigate the contribution of each test to the decision-making process, a model-agnostic explaining approach SHAP was introduced to determine the extent to which a feature or features of a task contributed to the diagnosis and severity evaluation. The analysis revealed that the alternative tapping test, circle drawing test and coordination test were highly important in the machine-learning process, with the alternative tapping test being the most crucial.

It draws our attention that among the three top important tests, the circle drawing test and the coordination test, are not directly covered in MDS-UPDRS. The circle drawing test is designed to detect micrographia^11^. Numerous studies have utilized digital tests to detect micrographia^13–17^, including the three versions of tests we incorporated in our design: spiral tracing, circle tracing, and circle drawing. While the spiral and circle tracing tests display a tracing cue on the screen, the circle drawing test challenges participants by requiring them to draw as large a circle as possible within a given screen area without any tracing assistance. Our results indicate that the circle drawing test is the most decisive among the three tests for micrographia detection.

The coordination test is designed to detect bimanual coordination^18^. Currently, plenty of clinical studies have demonstrated that PD results in impairments in bimanual movement, which involves the simultaneous left- and right-hand symmetrical movement (in-phase) or asymmetrical movement (anti-phase)^18–21^. However, there is no existing digital test for detecting bimanual coordination in PD patients. One advantage of using a digital platform for this test is the high sensitivity it offers in capturing detailed spatiotemporal information of finger movements. We innovatively designed a test by placing two symmetric trails on the left and right sides of the screen and requiring the test subjects to trace the two trails with their two index fingers in a synchronized fashion. The sensitivity of the digital platform in collecting spatiotemporal information of finger movements makes it an ideal tool for detecting bimanual coordination key indications, such as the difference between the left and right finger trajectories and bias between the drawn trail and the displayed trail, as demonstrated by our results. The importance of digitized coordination tests is consistent with clinical findings in non-digital settings.

It is worth mentioning that the alternative tapping test, which is demonstrated as the most decisive test in PDMotion, is designed differently from the original tapping test in MDS-UPDRS Part III. It requires the test subjects to tap alternatively on two circles displayed on the screen with their index fingers and middle fingers as fast as they can, rather than tapping their index fingers and thumbs in the air. The design greatly enables the platform to record the accurate and exact time and position information of both valid taps (paired taps inside the circles) and invalid taps (no paired taps or taps outside the circle).

To further confirm the validity of the alternative tapping test, we conducted experiments by only employing features extracted from it to machine learning models. The findings indicate that using the alternative tapping test alone produces comparable results for diagnosis and severity evaluation, when compared to using features from all tasks. This highlights the potential of the alternative tapping test as a quick and effective standalone mobile screening test.

The importance of the three tasks indicates that tasks that are not identical to those in the MDS-UPDRS Part III play a critical role on a digital platform. Digital platforms provide the ability to capture more precise and accurate information that may not be easily obtainable through traditional paper-and-pencil tests, such as spatiotemporal and sensory information, making them more suitable for detecting certain indications of PD motor symptoms.

There were still several limitations in the current study. First, subjects were recruited in a single center. Besides, the label classes were highly imbalanced. The number of subjects with advanced stages of the disease (H&Y ≥ 4, MDS-UPDRS Part III > 58) was relatively small. Considering that the study was conducted during the COVID-19 era, such patients with physical restraints may have taken virtual visits instead of in-person visits, thus less likely to be screened to participate in this study. Moreover, some of HCs had MDS-UPDRS Part III scores that were close to the scores of the patients with mild PD. MDS-UPDRS is designed to be rated as what is observed. Elevated MDS-UPDRS scores are common among individuals in the general population who do not have parkinsonism, particularly among older age groups, women, and those with specific comorbidities^9^. Accordingly, healthy elderly with decreased physical activities can be rated as high as 32. However, such noisy nature of the MDS-UPDRS as a label is the inherent limitation of the manually rated system. It is the rationale behind this study and related research to develop a more objective, digitalized biomarker for the disease. In addition, due to time constraints and the COVID-19 situation, we only recruited 79 participants, making our current dataset relatively small. To address these issues, we plan to collect larger data through an international, multi-centered design in the long term to improve the generalizability of our model.

Our study is the first to investigate the importance of different tests on a digital platform in the PD prediction process. We built the task importance analysis on top of a thorough coverage of motor tests, accurate machine learning models, and a reliable model-agnostic explainable approach. Our analysis revealed that the alternative tapping test, circle drawing test, and coordination test were the most important tasks, with the alternative tapping test being identified as the most critical for the machine learning process. Further experiments demonstrated that the alternative tapping test alone can be used as a rapid and effective standalone mobile screening test, as its performance was comparable to the performance of the entire test set.

## Methods

**Figure 6:**
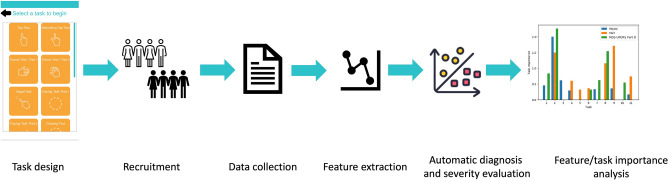
PDMotion method design pipeline.

In this section, we elaborate on the PDMotion pipeline from the following six aspects: task design, recruitment, data collection, feature extraction, automatic diagnosis and severity evaluation, and feature/task importance analysis as shown in Fig. 6. The task design subsection describes the inspiration and medical grounds for designing the assessment tasks, the design decisions in the digitalization process, and the detailed instructions and interfaces of tasks in PDMotion. The recruitment section describes detailed information on the recruitment criteria. The data collection part outlines key procedures for gathering the medical and demographic backgrounds of the participants and the data from PDMotion. The feature extraction subsection describes the features we extract in each task from the collected data. The automatic diagnosis and severity evaluation subsection describes the overall approach to classify the participants into different severity classes of PD or predict a score reflecting the PD severity level. Lastly, the feature/task importance analysis subsection introduces the explanation model used to identify the important features and tasks.

### Task design

We proposed a mobile platform PDMotion, aiming to capture motor symptoms of PD and support automatic motor examination in a digitalized way. A large part of PDMotion was designed to incorporate the assessments in the motor section of the MDS-sponsored Revision of the Unified Parkinson’s Disease Rating Scale (MDS-UPDRS)^22^ in a self-administrable and user-friendly way. MDS-UPDRS contains four parts to evaluate the various aspects of PD and is widely used in both clinical and research settings. Part III of MDS-UPDRS includes motor examinations that assess the motor signs of PD, such as tremor tests, finger and foot tapping, and gait assessment. To augment the examinations in MDS-UPDRS Part III, we also designed some novel tasks to capture PD motor symptoms that are not covered in MDS-UPDRS, including micrographia^11^ and bimanual coordination^18^.

PDMotion contains 11 assessment tasks. As shown in Table 3, the tasks can be divided into three types, tapping tests, tracing/drawing tests and accelerometer-related tests. Tapping tests include single tapping test, alternative tapping test, and target test for finger tapping, and foot tapping test. Tracing/drawing tests include spiral tracing test, circle tracing test, drawing test, and coordination test. Accelerometer-related tests include tremor tests (rest and postural) and gait test.

**Table 3:** The assessed PD symptoms and matching clinical or experimental evidence for the 11 assessment tasks.

Test	Assessed PD motor aspects	Clinical or experimental evidence
Task 1. Single tapping test	Bradykinesia of upper extremities	MDS-UPDRS Part III 3.4 tapping test Alternative finger tapping^23^
Task 2. Alternative tapping test		
Task 5. Target test		
Task 6. Circle tracing test	Micrographia	Standardized handwriting for assessment of micrographia
Task 7. Spiral tracing test		
Task 8. Circle drawing test		
Task 9. Coordination test	Finger coordination	Bimanual coordination in Parkinson's disease^18^
Task 3. Rest tremor test	Rest tremor severity	MDS-UPDRS Part III 3.17 rest tremor amplitude
Task 4. Postural tremor test	Postural tremor severity	MDS-UPDRS Part III 3.15 postural tremor of the hands
Task 10. Foot tapping test	Bradykinesia of the lower extremities	MDS-UPDRS Part III 3.7 toe tapping
Task 11. Gait test	Gait speed	MDS-UPDRS Part III 3.10 gait

**Figure 7:**
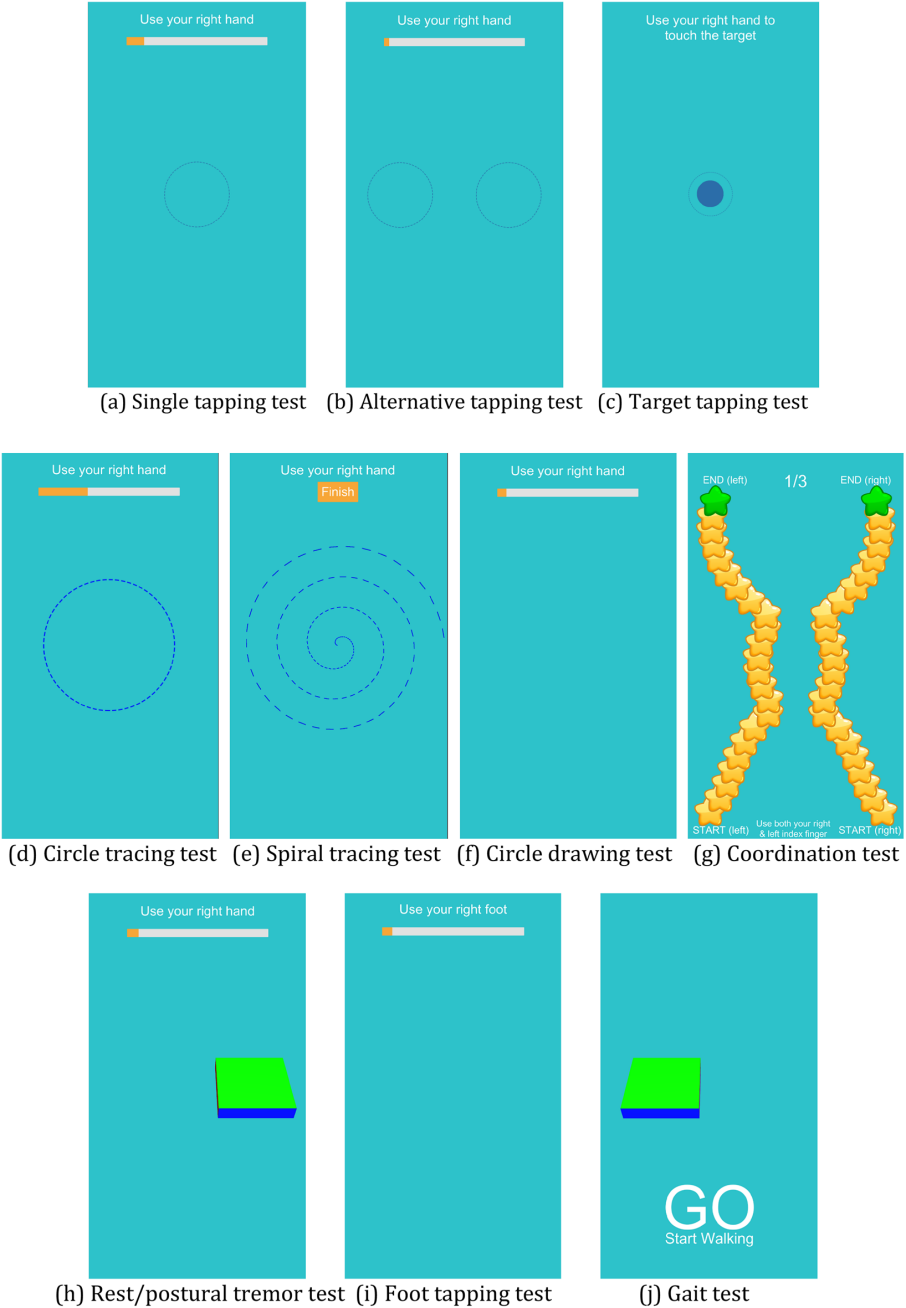
The interface of 11 tests.

The finger tapping tests (Fig. 7a–c) are digitalized from MDS-UPDRS Part III 3.4 tapping test. These tests require test subjects to tap within the circles displayed at the screen center with specific requirements. Each hand is assessed separately. The single tapping test requires the subject to perform one-finger tapping with an index finger at the centrally displayed circle, while the alternative tapping test requires alternate tapping with index and middle fingers^23^ on two circles as quickly as possible. The target tapping test requires the test subject to alternatively tap the tip of the nose and the target on the screen at the fastest speed.

Tracing/drawing tests (Fig. 7d–g) require the test subject to draw or trace given patterns with an index finger. The spiral tracing test, circle tracing test and circle drawing test are designed to screen for micrographia^11^, while the coordination test is for screening bimanual coordination in fine motor control^12,18^. Spiral and circle drawing was first applied to assess micrographia in PD^13^. In the spiral tracing test and the circle tracing test, the test subject is instructed to use one index finger to trace the spiral (once) or circle (multiple times) displayed on the screen. The drawing test elevates the difficulty level by removing the tracing cue and requiring the test subjects to draw circles as large as they can within the screen area. Research in Ref.^12^ provided evidence for impaired finger coordination in fine motor control of PD patients. The coordination test displays two symmetric trails on the left and right sides of the screen. The test subjects are required to trace the two trails with their two index fingers in a synchronized fashion.

Accelerometer-related tests (Fig. 7h–j) include those that rely on built-in accelerometers to collect motion data. The rest tremor test and postural tremor test are digitalized from MDS-UPDRS Part III 3.17 rest tremor amplitude and 3.15 postural tremor of the hands, respectively, to assess the severity level of tremor in PD. The foot tapping test is adapted from MDS-UPDRS Part III 3.7 toe-tapping, while the gait test corresponds to MDS-UPDRS Part III 3.10 gait. During the two tremor tests, the test subject is required to attach the device to the back of each hand using a hand strap. In the rest tremor test, the participant is instructed to sit still and rest the palm of the tested hand on the lap; while in the postural tremor test, the participant is asked to outstretch both arms in front of him/her with palms facing down. For the foot tapping test and gait test, the test subject is required to tie the phone around his/her ankle. The foot tapping test requires the test subject to tap the heel against the ground as fast and as consistently paced as he/she can. In the gait test, the test subject is instructed to perform the following sequence of actions: (1) stand up from the chair, (2) walk straight to a 3-m mark on the floor, (3) turn around to the right until facing the chair again, (4) walk back to the chair and (5) take a seat. The phone-embedded accelerometer tracked the motor information in real time.

During the digitalization process, some of the tests can be easily and directly converted into digital forms from the MDS-UPDRS Part III. For example, the postural tremor examination is digitalized by requiring the participant to attach the phone to the back of the hand while stretching the arms out in front of the body with palms down, the same actions as instructed in MDS-UPDRS Part III. Meanwhile, some tests are more challenging to digitize as they cannot be directly converted into a phone-administrable form. For example, the finger tapping in MDS-UPDRS Part III requires the test subject to tap the index finger on the thumb tip using the tested hand as quickly as possible. However, it is difficult to capture valid finger taps using smartphones without fingers touching the screen. Therefore, we adapted the MDS-UPDRS finger tapping into a single tapping test and an alternative tapping test to facilitate data collection on smartphones. Some examinations in MDS-UPDRS Part III were not covered in our platform, as they cannot be self-administered or considered unsafe without the supervision of caregivers or clinicians. Taking MDS-UPDRS 3.12 postural stability as an example, it requires a clinician to pull on the shoulders of the subject, which needs assistance and may lead to falling hazards.

Since certain motor symptoms of PD, e.g., micrographia and bimanual coordination, are not evaluated in MDS-UPDRS Part III, we designed tracing and drawing tests based on existing literature on PD motor assessment to increase coverage for these symptoms. With the eleven assessment tasks described above, PDMotion aims to provide a comprehensive platform for assessing motor symptoms and measuring the severity of PD.

### Recruitment

Participants who were diagnosed as PD according to the United Kingdom Parkinson’s Disease Society Brain Bank Criteria by movement disorder specialists were included. Exclusion criteria included age under 19 or above 85, diagnosis of atypical Parkinsonism, and history of any other neurological disease diagnosed by their treating neurologists (e.g., stroke), and significant cognitive impairment as defined by MoCA below 16 that may affect the task performances. The same exclusion criteria were applied to the recruitment of HCs. All participants were recruited from the Pacific Parkinson’s Research Centre, University of British Columbia. All patients have provided written informed consent. The study protocol was approved by the Institutional Review Board of The University of British Columbia (approval number: H16-02454) and of Nanyang Technological University (approval number: IRB-2018-12-010). The research was performed in accordance with relevant guidelines and regulations.

### Data collection

All study subjects first underwent a clinical evaluation, followed by assessments with the PDMotion. The subjects’ interactions with PDMotion were also supervised and guided by medical professionals.

Each subject was evaluated by three scales, MDS-UPDRS Part III^22^, H&Y^24^, and MoCA^25^.

- The MDS-UPDRS Part III motor examination contains 18 questions, and each question is scored on a 0–4 rating scale, indicating the severity of normal, slight, mild, moderate, and severe, respectively.
- H&Y is a five-stage rating scale of functional disabilities associated with PD progression, where stage 1 is the mildest stage and stage 5 is the severest stage.
- MoCA is a screening instrument for mild cognitive impairment that examines various cognitive domains. MoCA was included to ensure that the recruited study subjects are cognitively intact enough to understand and perform the tasks included in PDMotion.

Other than the three medical rating scales, additional information, such as age, gender, dominant hand, dominant foot, and medication status, was recorded as well. After undergoing the clinical evaluation, all test subjects were required to complete the assessment tasks in the PDMotion. Data were collected during the test process and uploaded to our cloud server for processing and analysis. The collected data is anonymized and falls into two broad categories:

- spatiotemporal data that records finger movements on screen, including time-stamped finger positions, collected from tapping, drawing and tracing tests, and
- accelerometer data obtained from phone-embedded sensors, including Euler angles and acceleration, collected from tremor and gait tests.

### Feature extraction

Rest tremor, bradykinesia, rigidity and loss of postural reflexes are four cardinal signs of PD^10^. Other motor features like micrographia, festination, shuffling gait and freezing are also considered characteristic clinical signs of PD^10^. In order to capture these motor signs, we extracted features for capturing salient PD symptoms as mentioned in Table 3. The extracted features and their matching tasks are illustrated in Table 4.

**Table 4:** Extracted features for each test.

	Test	Extracted features
1	Single tapping test	Tapped (total taps, valid taps), missed, bias, tapping frequency (mean value and variance of tapping intervals, tapping frequency declining slope from start to end)
2	Alternative tapping test	Tapped (total taps, valid taps, paired taps), missed, bias, tapping frequency (mean value and variance of tapping intervals, tapping frequency declining slope from start to end)
3	Rest tremor test	Rotation, acceleration, tremor frequency
4	Postural tremor test	Rotation, acceleration, tremor frequency
5	Target test	Duration, tapped (total taps, valid taps), tapping frequency (mean value and variance of tapping intervals, tapping frequency declining slope from start to end)
6	Circle tracing test	Bias, tracing velocity (average tracing velocity, tracing velocity declining slope from start to end), reduction slope of distance between finger position and circle centroid
7	Spiral tracing test	Bias, tracing velocity (average tracing velocity, tracing velocity declining slope from start to end), reduction slope of distance between finger position and spiral centroid
8	Circle drawing test	Bias, drawing velocity (average drawing velocity, drawing velocity declining slope from start to end), reduction slope of distance between finger position and circle centroid
9	Coordination test	Bias, tracing velocity (average tracing velocity, tracing velocity declining slope from start to end), left- and right-hand position coordination, passed trail points
10	Foot tapping test	Tapped
11	Gait test	Walking velocity, total steps

For all finger tapping tests, tapped and missed refer to the number of taps within the valid area (inside the circle) and invalid area (outside the circle), respectively. Bias refers to the sum of the distance between the tapping locations and the centroid of the circle. A smaller bias indicated that the tapping locations are more precise. Tapping frequency is reflected by the number of taps performed in a given time. The tapping frequency declining slope from start to end was calculated to indicate the change of frequency in finger movement. A decreasing tapping frequency may be an indicative sign of bradykinesia^26^.

In the tests that require test subjects to trace or draw a circle or a spiral, their drawings are characterized by the change in the distance between the finger position and the centroid of the circle or the spiral. A reduction of distance between finger position and circle centroid could be an indicative sign of micrographia^13^, which is manifested as an abnormal reduction in writing size and is a specific behavior deficit associated with PD^27^. The finger positions on the screen were tracked three times per second and the distance between the finger and the circle/spiral centroid was recorded as a serial ${r}_{1}, {r}_{2}, \dots , {r}_{n}$. We applied a simple linear model $r=at+b$ to fit the relationship between the distance and time. The slope $a$ can reflect the diminishing trend of the hand drawing. In tests that require test subjects to trace a trail, bias is used to measure the difference between the trail drawn by the user and the trail displayed on the screen. Impaired finger control ability may lead to a larger bias. Left- and right-hand position coordination was calculated by recording the difference between left- and right-hand positions. Poor bimanual coordination in tasks involving the simultaneous performance of either symmetrical or asymmetrical movements with both hands is a very early sign of PD^19^.

For tremor tests, we measured the rotation and the acceleration of the device in different directions to measure the motor changes. In addition, we applied the Fast Fourier Transform (FFT) to convert the sensor data from the spatial domain to the frequency domain and calculate the frequency distribution. We used the weighted average of FFT decomposed frequencies to approximate the tremor frequency. For the foot tapping test and gait test, we located the maxima of acceleration in different directions of accelerometer data to count taps or steps.

### Automatic diagnosis and severity evaluation

With the extracted features, we focused on two problems: automatic diagnosis and severity evaluation. Specifically, automatic diagnosis is treated as a bi-class classification problem that distinguishes PD patients from HCs. Severity evaluation is treated as a multi-class classification problem that classifies the test subjects into different groups based on their PD severity levels.

As mentioned in the Data Collection part, we adopted three medical rating scales, including MDS-UPDRS Part III, H&Y and MoCA. MoCA, the cognitive evaluation scale, is not for evaluating the severity of PD and hence is not used as a classification criterion in our design. H&Y is a class-based rating scale whose ratings serve as natural classes for the severity evaluation problem. MDS-UPDRS Part III, however, is a score-based rating criterion. In order to partition test subjects into groups based on their MDS-UPDRS Part III performance, we followed the cut-off scores proposed in Ref.^8^ to divide the PD patients into three motor severity ranges (mild, moderate, and severe) based on their MDS-UPDRS Part III scores. The cut-off points between mild/moderate and moderate/severe levels were 32/33 and 58/59, respectively.

We leveraged the aforementioned classification criteria and focused on one binary and two multi-class classification problems that classify study subjects as follows:

1. HCs and PD patients (2 classes in total)
2. HCs and PD patients categorized by H&Y (stage 1–5 for different severity levels for PD patients; 6 classes in total)
3. HCs and PD patients categorized by MDS-UPDRS Part III (severity levels mild, moderate and severe for PD patients as proposed in Ref.^8^; 4 classes in total)

For the classification algorithms, we applied classification models including single-layer linear neural network, SVM, logistic regression, random forest and XGBoost (eXtreme Gradient Boosting) to generate automatic diagnosis and severity evaluation.

### Feature/task importance analysis

From a machine learning perspective, it is important to analyze the input–output relationships for future model improvement. Specifically, in our applicational background, feature and task importance analysis can provide useful insights to guide task selection or future designs of PD digital screening platforms.

We adopted SHAP (SHapley Additive exPlanations)^28^ for feature and task importance analysis, which is a unified approach for quantifying the degree to which each feature contributes to the predictive power of a model. SHAP draws its inspiration from cooperative game theory and follows an additive feature attribution method, i.e. the output of the model is viewed as a linear addition of inputs. It computes the Shapley value^29^ for each feature, which can be interpreted as a measure of the contribution of a feature toward the model predictions. SHAP is capable of quantitatively explaining how each feature affects the prediction of the model for the entire dataset (global explanations) and for a given individual data sample (local explanations). In our paper, we are more concerned with global explanations as we are more interested in how the model’s predictive power is affected on the dataset scale.

SHAP specifies the explanation $g(x{\prime})$ as:

$$g\left({x}{\prime}\right)={\varphi }_{0}+ \sum_{j=1}^{M}{\varphi }_{j}{x{\prime}}_{j}$$

where $g$ is the explanation model, $x{\prime}\in {\{\mathrm{0,1}\}}^{M}$ is the vector of simplified input variables where 1 means the corresponding feature value is “present” and 0 means it is “absent”, $M$ is the number of features, ${\varphi }_{0}$ is a constant when all inputs are absent, and ${\varphi }_{j}\in {\mathbb{R}}$ is the feature importance attribution (i.e., the Shapley value) for feature $j$.

**Figure 8:**
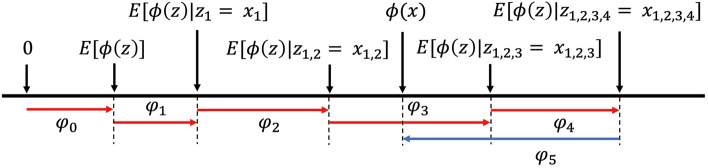
Interpreting Shapley values.

Shapley values can be used to quantify the contribution of a feature to the model output^30^. As shown in Fig. 8, consider the Shapley value for five features ${x{\prime}}_{j\in \{\mathrm{1,2},\mathrm{3,4}, 5\}}$. Without the presence of any of the five features, the average prediction $E[\phi (z)]$ is set at ${\varphi }_{0}$. ${\varphi }_{j\in \{\mathrm{1,2},\mathrm{3,4}, 5\}}$ represents the Shapley values of each feature. With their contributions, the prediction arrives at the final value $\phi (x)$. Note that Shapley values are additive. Shapley values can be positive and negative as features may contribute to model predictions in different directions. Consider the current example in a bi-class classification problem with class A and class B, assuming features 1, 2, 3 and 4 are pro class A and against class B as ${\varphi }_{1},{\varphi }_{2},{\varphi }_{3}, {\varphi }_{4}$ have the same sign (positive, illustrated in red). Then, feature 5 is pro class B and against class A as ${\varphi }_{5}$ has a different sign (negative, illustrated in blue) from the rest. The magnitude of ${\varphi }_{j}$ indicates the size of the effect.

### Literature review

In this section, we present a comprehensive overview of the current state-of-the-art mobile platforms for PD prediction and compare them with our work. This includes an analysis of task design and coverage and assesses the explainability of these platforms.

Table 5 summarizes studies that employ digital tasks via mobile phones to identify motor symptom features of Parkinson's Disease (PD). Studies that primarily focus on task design and feature distribution analysis between PD and healthy control groups^31–34^ are not included, as their scope does not encompass predictive modelling. Conversely, this table highlights research^3–7,35^ that utilizes motor features to predict PD status or severity. Importantly, tests indicated in bold represent digital tests that are designed originally in digital form and do not have a paper-and-pencil counterpart in the MDS-UPDRS Part III. These novel tests merit particular attention as they exploit the precision of mobile platforms in capturing nuanced movements, potentially identifying symptoms that are subtle and less apparent. Furthermore, these novel tests may uncover symptoms not covered by the MDS-UPDRS Part III, thereby broadening the scope of PD detection.

**Table 5:** Comparative overview of mobile PD prediction platforms.

	^5^	^4^	^6^	^3^	^7^	^35^	Ours
Task coverage							
Single tapping test							√
Alternative tapping test		√	√		√	√	√
Rest tremor test	√						√
Postural tremor test							√
Target test							√
Voice test			√		√		
Balance test			√		√		
Reaction time			√		√		√
Circle tracing test							√
Spiral tracing test		√		√			√
Circle drawing test							√
Coordination test							√
Foot tapping test							√
Gait test	√		√		√		√
Explainability							
Feature importance analysis		√	√	√			√
Task importance analysis			√				√
Top feature/task effectiveness validation							√

From Table 5, it can be observed that the alternative tapping test emerges as the most prevalently used digital test, possibly attributed to its ease of implementation. The gait test also stands out as a frequently used and well-researched test, because abnormal gait is one of the most significant indicators of PD^36^, and gait abnormalities have been linked to disease severity^37^. Studies^6,7^ designed voice and balance tests, which are not included in our PDMotion application because they pertain to the MDS-UPDRS Part II: Motor Aspects of Experiences of Daily Living (M-EDL), focusing on long-term monitoring outside the direct motor examination scope of our study. Compared to other PD mobile-assessment platforms, PDMotion distinguishes itself by offering the broadest array of tests, which include finger tapping tests, tremor tests, and the spiral tracing test. Additionally, PDMotion contains five novel digital tests which have not been used and studied in other similar platforms, i.e., the target test, the circle tracing and drawing, the coordination test, as well as the foot tapping test. This broad coverage of assessments enables a comprehensive study and comparison of the efficacy of various digital tests in the context of Parkinson's Disease.

For PD mobile assessment platforms, in addition to improving accuracy, considerable research efforts have been dedicated to feature/task importance analysis to enhance explainability. Feature importance analysis can reveal motor symptom features that are most pivotal in predictive modelling. Task importance analysis can help to evaluate the efficacy of various digital tests. These analyses can contribute significantly to our understanding of PD symptomology and inform the future design and implementation of digital assessment tools for PD. Therefore, this section also reviews the explainability aspect of different PD mobile-assessment platforms, more specifically, reviewing whether these platforms include feature/task importance analysis with subsequent validation to enhance explainability. Among the studies focused on PD prediction, study^3^ applies the Mann–Whitney U Test for statistical comparison of feature distributions between patients and control groups. However, this approach does not elucidate the features that actually influence the prediction-making process. In study^4^, six out of 17 motor skill test features were identified as significant for a regression model, but the use of Principal Component Analysis (PCA) leads to a loss of explainability. Moreover, these features are assessed against a regression model, which did not emerge as the most effective among the four machine learning models employed in the study. Study^6^ engages a rank-based algorithm for deriving disease severity scores to ascertain feature importance weights. Notably, this study^6^ also draws a task-level insight on the most important task in PD prediction on a mobile platform. It highlights the gait test as a key experimental measure by aggregating feature importance scores. However, it falls short in validating the gait test's effectiveness as a standalone diagnostic tool.

PDMotion distinguishes itself by rigorously examining the influence of a variety of digital tests within the framework of PD prediction. This exploration into task relevance is underpinned by a comprehensive coverage of motor tests and supported by a reliable model-agnostic explainable approach. Crucially, the results derived from the task importance analysis shed light on key features that are instrumental in the accurate prediction of PD. These findings are not only pivotal for the understanding of PD but also provide invaluable guidance for the future design and implementation of digital assessment tools in this domain.

## Data Availability

The data that support the findings of this study are available from the corresponding author upon reasonable request.

## Abbreviations

FFT: Fast Fourier Transform
HC: Healthy control
H&Y: Hoehn and Yahr Scale
MDS-UPDRS: MDS-sponsored Revision of the Unified Parkinson’s Disease Rating Scale
MoCA: Montreal Cognitive Assessment
PCA: Principal Component Analysis
PD: Parkinson’s disease
SHAP: SHapley Additive exPlanations
XGBoost: Extreme Gradient Boosting

## Code availability

The analysis code of this study is available from the corresponding author upon reasonable request.
